# JDP2 is directly regulated by ATF4 and modulates TRAIL sensitivity by suppressing the ATF4–DR5 axis

**DOI:** 10.1002/2211-5463.13017

**Published:** 2020-11-13

**Authors:** Máté János Engler, Junsei Mimura, Shun Yamazaki, Ken Itoh

**Affiliations:** ^1^ Department of Stress Response Science Center for Advanced Medical Research Hirosaki University Graduate School of Medicine Japan

**Keywords:** ATF4, cancer, DR5, JDP2, TRAIL

## Abstract

Jun dimerization protein 2 (JDP2) is a bZip‐type transcription factor, which acts as a repressor or activator of several cellular processes, including cell differentiation and chromatin remodeling. Previously, we found that a stress‐responsive transcription factor, known as activating transcription factor 4 (ATF4), enhances *JDP2* gene expression in human astrocytoma U373MG and cervical cancer HeLa cells; however, the role of JDP2 in the ATF4‐mediated stress response remained unclear. Here, we reported that siRNA‐mediated JDP2 knockdown enhances the expression of several ATF4 target genes, including *ASNS,* and death receptors 4 and 5 (*DR4* and *DR5*) in HeLa cells. In addition, the results of a transient reporter assay indicate that JDP2 overexpression represses ER stress‐mediated DR5 promoter activation suggesting that JDP2 negatively regulates ATF4‐mediated gene expression. Curiously, knockdown of JDP2 increases the sensitivity of cells to TNF‐related apoptosis‐inducing ligand (TRAIL), which induces apoptosis in cancer cells through DR4 and DR5. These results indicate that JDP2 functions as a negative feedback regulator of the ATF4 pathway and contributes to TRAIL resistance in cancer cells.

## Graphical abstract

Here, we found transcriptional repressor JDP2 is transcriptionally induced by ATF4 via AARE in the *JDP2* promoter. JDP2 functions as a negative feedback regulator of the ATF4 pathway and suppresses ATF4‐mediated death receptor DR5 expression. JDP2 suppresses TRAIL‐mediated apoptosis by inhibiting the ATF4‐DR5 axis.

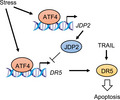

**Abbreviations**
ATF4: activating transcription factor 4
DR5: death receptor 5
JDP2: jun dimerization protein 2
Tg: thapsigargin
Tm: tunicamycin
TRAIL: TNF‐related apoptosis‐inducing ligand

Jun dimerization protein 2 (JDP2) is a bZip‐type transcription factor that belongs to the activator protein‐1 (AP‐1) family [1]. JDP2 was initially described as a repressor of AP‐1, but it also functions as an activator in different cellular contexts. JDP2 suppresses 12‐*O*‐tetradecanoylphorbol‐13‐acetate (TPA) response element‐dependent transcription or cAMP‐responsive element‐dependent transcription by heterodimerizing with Jun family proteins, such as c‐Jun and JunB [1, 2]. JDP2 epigenetically silences gene expression by recruiting histone deacetylases, such as histone deacetylase 3 [1, 3]. However, JDP2 also functions as a coactivator of progesterone receptor and NF‐E2‐related factor 2 (Nrf2) [4, 5]. JDP2 knockout mice display abnormal osteoclast and neutrophil differentiation [6, 7]. Furthermore, JDP2 suppresses adipocyte differentiation, thus suggesting that JDP2 has an essential role in cell differentiation [8]. Previously, we identified *JDP2* as a gene that is upregulated by carnosic acid (CA), which is an ingredient of western herb rosemary that activates activating transcription factor 4 (ATF4) [9]. Other studies also suggested that ATF4 regulates *JDP2* gene expression [10, 11]. However, the precise mechanism by which ATF4 activates *JDP2* expression remains unknown.

ATF4 is a stress‐responsive transcription factor that plays an important role in the integrated stress response [12]. ATF4 is activated in response to various stresses, such as endoplasmic reticulum (ER) stress and amino acid deficiency, and modulates the expression of target genes. ATF4 transactivates gene expression by binding to its recognition element, namely the amino acid response element (AARE) (the consensus sequence is TGATGnAAn, where n is any nucleotide) [12]. ATF4 upregulates several cytoprotective and stress response genes, such as those involved in amino acid metabolism and the unfolded protein response [12]. ATF4 also induces apoptosis by enhancing C/EBP homologous protein (CHOP) and death receptors 4 and 5 (DR4 and DR5) in response to stress [13, 14]. CHOP activates proapoptotic factors, such as B‐cell lymphoma‐2 (Bcl‐2)–like protein 11 and p53 upregulated modulator of apoptosis, but suppresses antiapoptotic factors, such as Bcl‐2, thus leading to intrinsic mitochondria‐dependent apoptosis [13]. DR4 and DR5 are receptors for TNF‐related apoptosis‐inducing ligand (TRAIL) and mediate TRAIL‐dependent extrinsic apoptosis in target cells and the Fas/Fas ligand system [14, 15]. TRAIL also induces TRAIL ligand‐independent apoptosis during glucose starvation or ER stress [16, 17]. TRAIL is mainly produced by immune cells as a membrane‐bound protein or a secreted protein that leads to caspase‐8‐dependent apoptosis [18]. Considering that DR4 and DR5 are expressed in tumor cells but not in normal cells, TRAIL and the death receptor system play an important role in tumor cell elimination by regulating the immune system. However, many tumor cells escape TRAIL‐mediated apoptosis by downregulating death receptors, overexpressing decoy receptors that lack a cytosolic domain, or overexpressing FLICE‐like inhibitory protein (c‐FLIP), which is a negative regulator of death receptor signaling [19]. Several transcription factors, including ATF4 and CHOP, are known to modulate *DR4* and *DR5* gene expression. However, the precise mechanism by which DR4 and DR5 are downregulated in tumor cells remains poorly understood.

## Materials and methods

### Reagents

Tunicamycin (Tm), dimethyl sulfoxide (DMSO), and recombinant TRAIL were obtained from Wako Pure Chemical Industries, Ltd. (Osaka, Japan). Thapsigargin (Tg) was purchased from Sigma‐Aldrich (St. Louis, MO, USA).

### Cell culture

U373MG cells were obtained from the European Collection of Cell Cultures. HeLa cells were purchased from the American Type Culture Collection. The cells were cultured in Dulbecco’s Modified Eagle’s Medium (Sigma‐Aldrich) supplemented with 10% fetal bovine serum and 1X penicillin/streptomycin (Life Technologies, Carlsbad, CA, USA). Cells were maintained at 37 °C in a 5% CO_2_ incubator.

### Reverse transcription quantitative PCR (RT‐qPCR)

Total RNAs were isolated using TRIzol reagent (Life Technologies) according to the manufacturer’s instruction. A PrimeScript II RT Kit (Takara Bio, Otsu, Japan) was used to synthesize cDNA. SYBR Premix EX Taq II (Takara Bio) and a CFX96 thermal cycler (Bio‐Rad, Hercules, CA, USA) were used for qPCR. The following primer pairs were used for the experiments: *JDP2* total, 5'‐ GAGGTGAAACTGGGCAAGAG‐3', and 5'‐ GCTGCTGCGACTTTGTTCTT‐3'; *JDP2* variant 2, 5'‐GGG AGG TTA AGG CTG GCC TG‐3' and 5'‐ AGC GTA TTT CAG CTC CA‐3'; *JDP2* variant 3, 5'‐CCT TCT GCA CGG CTG GCC T‐3' and 3' primer of variant 2; *DR5*, 5'‐ATC GTG AGT ATC TTG CAG CC‐3' and 5'‐TGA GAC CTT TCA GCT TCT GC‐3'; *DR4*, 5'‐GCT GTG CTG ATT GTC TGT TG‐3' and 5'‐TCG TTG TGA GCA TTG TCC TC‐3'; *c‐FLIP*, 5'‐TCT CAC AGC TCA CCA TCC CTG‐3' and 5'‐CAG GAG TGG GCG TTT TCT TTC‐3'; or described elsewhere [9].

### siRNA transfection

U373MG or HeLa cells were seeded into 12‐well plates (1 × 10^5^ cells per well). The next day, the cells were transfected with siRNA by using Lipofectamine RNAiMax Reagent (Life Technologies). After a 24‐h transfection, the medium was replaced with a fresh medium, and the cells were treated with the indicated drugs. The target sequence of human *ATF4* and *JDP2* are 5′‐ GCC TAG GTC TCT TAG ATG A‐3′ and 5′‐GTG AGC TAG ATG AGG AAG A‐3′, respectively. A negative control siRNA (cat. #1027310, Qiagen, Hilden, Germany) was used for negative control experiments.

### Immunoblot analysis

Immunoblot analysis was performed as described before [9]. Briefly, an aliquot of nuclear extract was subjected to sodium dodecyl sulfate–polyacrylamide gel electrophoresis and blotted with antibodies against ATF4 (CREB2) (C‐20) and Lamin B (M‐20) purchased from Santa Cruz Biotechnology (Dallas, TX, USA).

### Plasmid construction

The JDP2 expression plasmid was constructed by PCR cloning. *JDP2* cDNA was amplified using the primer pair 5'‐CGG GAT CCC CTG CTA TGA TGC CTG GGC‐3' and 5'‐ GGA ATT CTC ACT TCT TCT CGA GCT GCT C ‐3' and was digested with BamHI/EcoRI. The purified DNA fragment was subcloned into the BamHI/EcoRI sites of the pcDNA3‐FLAGx3 vector [9]. To construct hJDP2‐Luc, the human JDP2 promoter was amplified using the primer pair 5'‐GCC AGA TCT GTC AGT GGG TGT GAA GCG CC‐3' and 5'‐GCC AAG CTT GCA GAA GGT GCG GGG GGA‐3', and U373MG genomic DNA was used as a template. After BglII/StuI digestion, the DNA fragment was subcloned into the BglII/HindIII sites of the pGL3 vector (Promega, Madison, WI, USA). pJDP2‐mt‐Luc was generated using site‐directed mutagenesis with the following primers: 5'‐CTA TAT AGC CGG GGC GGG TGC AAC CCG TCC CGC‐3' and 5'‐GCG GGA CGG GTT GCA CCC GCC CCG GCT ATA TAG‐3'. For the construction of pDR5‐Luc, the human DR5 promoter was amplified using PCR primers (5'‐AAA CAA ACC ACA GCC CGG GGC GCA‐3' and 5'‐CTG TCC CCG TTG TTC CAT GGC GGT‐3') and subcloned into the SmaI/NcoI sites of the pGL3 vector.

### Transient transfection and reporter assay

HeLa cells were seeded into a 24‐well plate (5.0 × 10^4^ cells per well) and incubated overnight. The next day, the cells were transfected with 200 ng of the luciferase reporter and 10 ng of pRL‐TK or 2 ng of the pRL‐EF *Renilla* luciferase coreporter plasmid and 200 ng of the effector plasmid by using the FuGENE transfection reagent (Promega). After a 4‐h incubation, the medium was replaced with a fresh medium containing either DMSO or 2 μg·mL^−1^ Tm. After a 20‐h incubation, the cells were subjected to a luciferase assay. Luciferase activity was measured using Dual‐Luciferase Assay Reagent (Promega) according to the manufacturer’s protocol and was normalized to *Renilla* luciferase activity.

### Cell viability assay

Cell viability was evaluated using a CCK‐8 kit (Dojindo, Kumamoto, Japan) according to the manufacturer’s protocol. Absorbance at 450 nm was measured using a microplate reader (Bio‐Rad).

### Statistical analysis

Student’s *t*‐test or one‐way ANOVA with the Tukey–Kramer *post hoc* test was used to estimate the statistical significance of the differences between two or more groups. Differences between groups were considered statistically significant with *P* values < 0.05. All experiments were repeated at least three times, and the data are expressed as mean ± standard error (SE), and individual data points are shown as open circles.

## Results

### ATF4 regulates *JDP2* expression

**Fig. 1:**
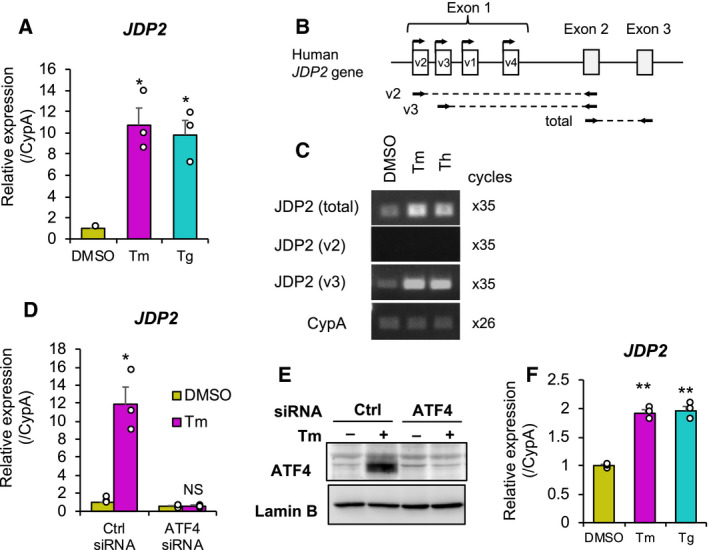
*JDP2* is induced by ATF4 activation. (A) U373MG cells were treated with DMSO (yellow bar), 2 μg·mL^−1^tunicamycin (Tm, magenta bar), or 1 μm thapsigartin (Tg,cyan bar) for 6 h, and *JDP2* expression was evaluated by RT‐qPCR as described in the Materials and Methods. The values were normalized to cyclophilin A (CypA) expression and were presented as mean ± SEM of three independent experiments (*n* = 3). Open circles indicate individual data points. (B) Schematic representation of the human *JDP2* gene locus; v1 to v4 indicate the alternative first exons of variants 1 to 4. The positions of specific primer pairs that amplify variant 2 (v2), variant 3 (v3), and total *JDP2* transcripts are described below. (C) The representative agarose gel electrophoresis image of amplified RT‐qPCR products using *JDP2* primer pairs, as indicated in (B). (D) *JDP2* expression in ATF4 knockdown cells. U373MG cells were transfected with siRNA. After a 24‐h transfection, the medium was changed, and cells were further incubated for 6 h in the presence of DMSO (yellow bar) or 2 μg·mL^−1^Tm (magenta bar). *JDP2* expression was determined similar to that in (A) and presented as mean ± SEM of three independent experiments (*n* = 3). (E) Representative immunoblot image of ATF4 knockdown cells. (F) HeLa cells were treated with DMSO (yellow bar), Tm (magenta bar), or Tg (cyan bar)for 6 h, and RT‐qPCR was performed. *JDP2* expression was determined similar to that in (A) and presented as mean ± SEM of three independent experiments (*n* = 3). The asterisks indicate the significant differences compared with the DMSO control (*P < 0.05; **P < 0.01; NS, not significant) by Student’s*t*‐test.

In a previous global transcriptome study, we found that *JDP2* is induced by CA, which is a phytochemical that activates both Nrf2 and ATF4 in U373MG human astrocytoma cells [9]. However, whether ATF4 modulates *JDP2* gene expression remains unknown. To investigate the role of ATF4 in the regulation of *JDP2* gene expression, we used the ATF4 inducers Tm and Tg, which activate ATF4 via ER stress in a PERK‐dependent manner. On the basis of RT‐qPCR analysis, we found that both Tm and Tg significantly induced *JDP2* gene expression in U373MG cells (Fig. 1A). The human *JDP2* gene has at least four alternative first exons (Fig. 1B). To identify the *JDP2* transcripts induced by ATF4 activation, we performed variant‐specific RT‐PCR by using the primer pairs shown in Fig. 1B. Given that variants 2 and 3 are highly expressed in human cells, we analyzed the expression of variants 2 and 3. Both Tm and Th specifically induced the transcript expression of *JDP2* variant 3 (Fig. 1C). We also found that the Tm induction of *JDP2* was completely abolished by ATF4 knockdown (Fig. 1D,E). Furthermore, both Tm and Th significantly induced *JDP2* in human cervical cancer HeLa cells (Fig. 1F). These results indicate that ATF4 modulates *JDP2* expression.

### The *JDP2* promoter is activated by ATF4

**Fig. 2:**
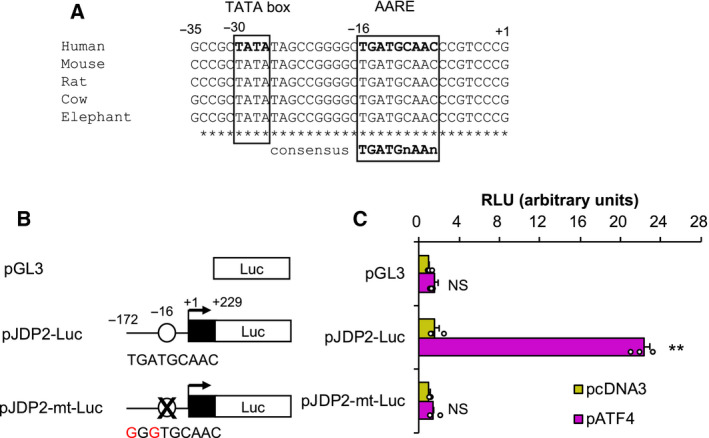
ATF4 activates the *JDP2* promoter via AARE. (A) Sequence alignment of the promoter region of mammalian *JDP2* genes. TATA box and AARE sequence are indicated. (B) Schematic illustration of the reporter gene constructs. AARE is represented by a blank circle. (C) *JDP2* promoter reporter gene activation by ATF4. HeLa cells were cotransfected with either an AARE intact (pJDP2‐Luc) or AARE mutant (pJDP2‐mt‐Luc‐mt) reporter plasmid, along with a vacant (pcDNA3, yellow bar) or ATF4 expression vector (pATF4, magenta bar). After a 4‐h incubation, the medium was replaced with fresh medium, and the cells were further incubated for 20 h. The luciferase activities were normalized with cotransfected *Renilla* luciferase activities and were presented as relative luciferase units (RLUs). The values are presented as mean ± SE of three independent experiments (*n* = 3). The asterisks indicate a significant increase compared with the pcDNA3 control (**P < 0.01; NS, not significant) by Student’s*t*‐test.

ATF4 regulates the expression of various target genes by binding to AAREs [12]. On the basis of in silico analysis, we found a consensus AARE sequence that is conserved between mammals immediately upstream of first exon of variant 3 of human *JDP2* gene (Fig. 2A). To confirm whether this AARE is responsible for the ATF4 induction of *JDP2*, we constructed a *JDP2* gene promoter‐luciferase plasmid and transfected it into HeLa cells with or without the ATF4 expression plasmid (Fig. 2B). Luciferase activity was significantly upregulated by ATF4 cotransfection (Fig. 2C). By contrast, no ATF4‐mediated induction was observed in the AARE‐mutated reporter construct (pJDP2‐mt‐Luc) (Fig. 2B,C). These results indicate that ATF4 regulates *JDP2* expression in an AARE‐dependent manner.

### JDP2 knockdown enhanced ATF4 and its target gene expression

**Fig. 3:**
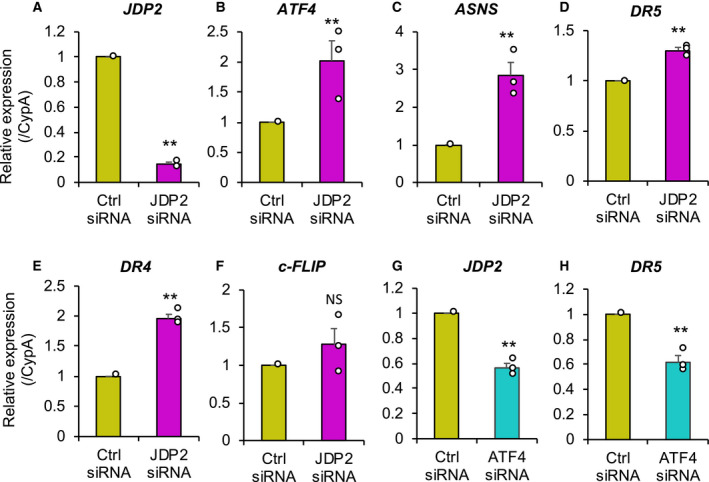
JDP2 knockdown enhanced *ATF4* and its target gene expression. HeLa cells were transfected with either control (Ctrl, yellow bar) or *JDP2* (magenta bar) or *ATF4* (cyan bar) siRNA. After 24‐h transfection, *JDP2* (A) and (G), *ATF4* (B), *ASNS* (C), *DR5* (D) and (H), *DR4* (E), and *c‐FLIP* (F) expression levels were evaluated by RT‐qPCR and normalized to CypA expression levels. The data are presented as the mean ± SE of three independent experiments (*n* = 3). The asterisks indicate a significant difference compared with control siRNA (**P < 0.01; NS, not significant) by Student’s*t*‐test.

To explore the physiological function of ATF4‐mediated *JDP2* induction, we performed JDP2 knockdown experiments by using siRNA. We confirmed by RT‐qPCR analysis that *JDP2* siRNA transfection considerably reduced *JDP2* expression in HeLa cells (Fig. 3A). *ATF4* mRNA expression was significantly upregulated by JDP2 knockdown (Fig. 3B). Furthermore, ATF4 target genes, such as *ASNS*, *DR5*, and *DR4*, were also significantly upregulated by JDP2 knockdown (Figs. 3C–E). Other ATF4 target genes, including *ATF3* and *CHOP*, were also substantially upregulated by JDP2 knockdown (data not shown). By contrast, the gene expression of *c‐FLIP*, which inhibits DR5 and DR4 signaling, was not affected by JDP2 knockdown (Fig. 3F). In addition, ATF4 knockdown significantly reduced *JDP2* and *DR5* expression in HeLa cells (Fig. 3G,H). These results indicate that JDP2 negatively regulates the ATF4 pathway.

### JDP2 negatively regulates TRAIL sensitivity

**Fig. 4:**
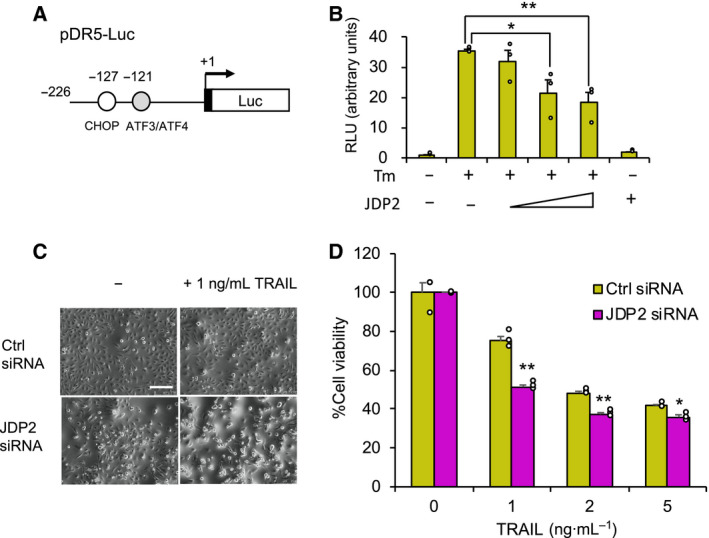
JDP2 decreases*DR5*promoter activity and affects TRAIL sensitivity. (A) Schematic illustration of the human*DR5*promoter reporter gene construct (pDR5‐Luc). (B) HeLa cells were cotransfected with the pDR5‐Luc reporter vector alone or with increasing amounts of the JDP2 expression plasmid as described. After a 4‐h incubation, the medium was replaced by fresh medium with or without 1 μg·mL^−1^Tm. After a 20‐h incubation, the luciferase activities were measured and normalized to*Renilla*luciferase activities. The RLUs are presented as mean ± SE. The asterisks indicate a significant difference compared with the control (*P < 0.05; **P < 0.01) by one‐way ANOVA with the Tukey–Kramer*post hoc*test (*n* = 3). (C) Representative images of control or JDP2 knockdown cells treated with 1 ng·mL^−1^recombinant TRAIL for 24 h. Scale bar indicates 250 μm. (D) Cell viability of TRAIL‐treated cells. Either control (yellow bar) or*JDP2*(magenta bar) siRNA transfected HeLa cells were treated with 1–5 ng·mL^−1^recombinant TRAIL. After a 24‐h incubation, cell viability was evaluated by the CCK‐8 kit. The relative cell viabilities (non‐treatment = 100%) are presented as mean ± SE. The asterisks indicate a significant difference compared with the control siRNA (**P < 0.01; *P < 0.05) by Student's*t*‐test (*n* = 3).

Given that DR5 plays an important role in TRAIL‐mediated cancer cell apoptosis, we next investigated whether JDP2 affects TRAIL sensitivity of cancer cells. Although DR5 expression was also induced by JDP2 knockdown in U373MG cells (Fig. S1A,B), U373MG cells show resistance to TRAIL by overexpression of c‐FLIP, that negatively regulates DR5 signaling (Fig. S1C) [20], we analyzed the role of JDP2 in the regulation of *DR5* gene expression and TRAIL sensitivity in HeLa cells. ATF4 modulates *DR5* gene expression by binding to the *DR5* gene promoter [21]. To investigate whether JDP2 affects *DR5* promoter activity, we performed reporter analysis by using a *DR5* promoter‐luciferase reporter construct (Fig. 4A). JDP2 overexpression decreased the activation of the *DR5* promoter induced by Tm in a dose‐dependent manner (Fig. 4B). Interestingly, although JDP2 knockdown did not decrease cell viability by itself, it significantly increased TRAIL sensitivity in HeLa cells (Fig. 4C,D). Although Tm induced endogenous *DR5* expression, further up‐regulation of *DR5* expression by JDP2 knockdown was not observed, although ATF4 knockdown down‐reduced *DR5* expression (Fig. S1D,E). On the other hand, JDP2 knockdown also increased TRAIL sensitivity of T98G human glioma cells (Fig. S2). These results indicate that JDP2 decreases TRAIL sensitivity, at least in part, by suppressing *DR5* gene expression.

## Discussion

In this study, we revealed for the first time that JDP2 decreases TRAIL sensitivity via the repression of an ATF4–DR5 axis. *DR5* gene regulation by ATF4 and its downstream targets ATF3 and CHOP is well established. ATF4 enhances the DR5‐dependent apoptosis of HeLa cells during glucose deprivation [14]. CHOP induces DR5 expression in response to Tg or fenretinide, which is a semisynthetic retinoid [22, 23]. Edagawa *et al*. [24] demonstrated that zerumbone and celecoxib activate ATF4 and downstream targets ATF3 and CHOP to induce *DR5* expression in human colorectal cancer cells.

In this study, we found ATF4/AARE‐mediated *JDP2* gene regulation (Figs 1 and 2). As shown in Fig. 2A, AARE in *JDP2* gene promoter are well conserved among mammals, suggesting its importance in *JDP2* gene regulation. We demonstrated that JDP2 knockdown substantially upregulated *DR5*, *ATF3*, *CHOP*, and *ATF4* expression (Fig. 3B,D, and data not shown) and that JDP2 negatively regulated ATF4‐mediated *DR5* gene promoter activity (Fig. 4B), thus indicating that JDP2 is a negative feedback regulator of the ATF4‐DR5 axis. JDP2 was reported to recruit multiple HDAC proteins to the *ATF3* promoter and suppress *ATF3* expression in HEK293Ts and MEFs [3]. Furthermore, JDP2 inhibits ATF4‐mediated *CHOP* induction in HeLa cells [21]. Therefore, it is likely that JDP2 downregulates *DR5* expression via the suppression of the ATF4–ATF3 and ATF4–CHOP axes. However, the mechanisms by which JDP2 suppresses ATF4 activity remain unknown. ATF4 interacts with multiple AP‐1 family proteins, including ATF3 and CHOP [25]. Among them, JDP2 is more similar to ATF3 in the bZip domain at the amino acid sequence level, which is responsible for dimer formation. Our preliminary study revealed that JDP2 interacts with ATF4 *in vitro* (data not shown), thus suggesting that JDP2 may suppress ATF4 activity by heterodimerizing with ATF4. Since Tm induction of *DR5* expression was not further enhanced by JDP2 knockdown (Fig. S2), JDP2‐mediated *DR5* gene regulation mechanism may be mediated by more complicated process. Further studies are needed to clarify this point.

In addition to ATF4, ATF3, and CHOP, *DR5* gene expression is regulated by multiple transcription factors, including AP‐1, FOXO3a, p53, and NF‐κB [26]. Given that JDP2 was first reported as an AP‐1 repressor, it is possible that JDP2 also inhibits AP‐1 activity to modulate *DR5* expression. Interestingly, Zou *et al*. [27] reported that c‐Jun N‐terminal kinase (JNK) upregulates *DR5* expression via AP‐1‐mediated CHOP induction. JNK is reported to phosphorylate JDP2 at Thr148 for proteasomal degradation [28], thus suggesting that JNK may regulate *DR5* expression by modulating not only AP‐1 but also JDP2 activities.

Although TRAIL selectively induces apoptosis in cancer cells, it is well known that several cancer cells develop TRAIL resistance via several mechanisms [19]. One mechanism is the downregulation of DR5 or DR4 by somatic mutation or epigenetic modification. Given that JDP2 is known as an epigenetic modulator, it may contribute to TRAIL resistance in cancer cells by suppressing DR4 and DR5 expression. However, it remains controversial whether JDP2 is a tumor suppressor or oncogenic factor. The inhibitory effect of JDP2 on oncogenic AP‐1 suggests that JDP2 may be a tumor suppressor. JDP2 inhibits the transformation of NIH3T3 cells by TPA, which activates AP‐1 [29]. Yuanhong *et al*. [30] reported that JDP2 downregulation is associated with metastasis in pancreatic cancer patients. It was also reported that JDP2 inhibits the epithelial–mesenchymal transition of pancreatic cancer BxPC3 cells [31]. By contrast, the present study suggests that JDP2 negatively regulates TRAIL/DR5 signaling and promotes tumor cell survival. It was reported that JDP2 overexpression enhances diethylnitrosamine‐induced liver cancer in mice [32]. The *JDP2* gene locus is activated by retroviral insertion in mouse T‐cell lymphomas [33]. Interestingly, JDP2 expression is suppressed by tumor suppressor p53, and this finding suggests the tumor promotion activity of JDP2 [34]. However, it is well established that many stress‐responsive transcription factors, such as ATF4, exhibit protective or antitumor activity in normal cells but support tumor cell survival under severe stress conditions [35]. This suggests that the function of JDP2 in tumor formation may change depending on physiological and pathological situations.

## Conclusions

In conclusion, this study shows that JDP2 functions as a negative feedback regulator of the ATF4 pathway and modulates TRAIL sensitivity in cancer cells. Further studies are needed to confirm whether JDP2 is a potential therapeutic target for TRAIL‐mediated cancer immunotherapy.

## Conflict of interest

The authors declare no conflict of interest.

## Author contributions

EMJ, JM, and KI designed and directed the project; EMJ, JM, and SY performed the experiments and analyzed the data. EMJ, JM, and KI wrote the manuscript. All authors discussed the results and commented on the manuscript.

## Supporting information


**Fig. S1.** Comparison of gene expression in either control or JDP2 knockdown HeLa and U373MG cells. Gene expression for *JDP2* (A), *DR5* (B) and *c‐FLIP* (C) was determined by RT‐qPCR and normalized with cyclophilin A expression. (D to F) HeLa cells were transfected with either control or JDP2 or ATF4 siRNA. After 24 h incubation, the media was replaced and treated with DMSO or 1 μg/mL tunicamycin for 6 h. RT‐qPCR was performed to evaluate *DR5* (D), *JDP2* (E) and *ATF4* (F) expression. The values are presented as mean ± SE of three independent experiments (n = 3).

**Fig. S2.** TRAIL sensitivity of T98G human glioma cells. T98G cells were transfected with either control or JDP2 siRNA by using Lipofectamine RNAiMAX. After 24 h transfection, the media was replaced with fresh media and incubated for 48 h in the absence or presence of TRAIL as indicated in figure. (A) Representative photo images of control or JDP2 knockdown cells treated with 5 μg/mL recombinant TRAIL for 48 h. Scale bar indicates 250 μm. (B) Cell viability was determined by CCK‐8 kit. The values are presented as mean ± SE of three independent experiments (n = 3) (‐TRAIL = 100%). The asterisks indicate a significant decrease compared with control (*, p < 0.05; NS, not significant) by Student’s t‐test.

## Data Availability

The additional data used to arrive at these conclusions can be obtained from the corresponding author on reasonable request.
