# Supplementary figures and images for: JDP2 is directly regulated by ATF4 and modulates TRAIL sensitivity by suppressing the ATF4–DR5 axis
Source: FEBS Open Bio. 2020 Nov 13;10(12):2771–9. doi: 10.1002/2211-5463.13017 (PMC7714084; doi:10.1002/2211-5463.13017)

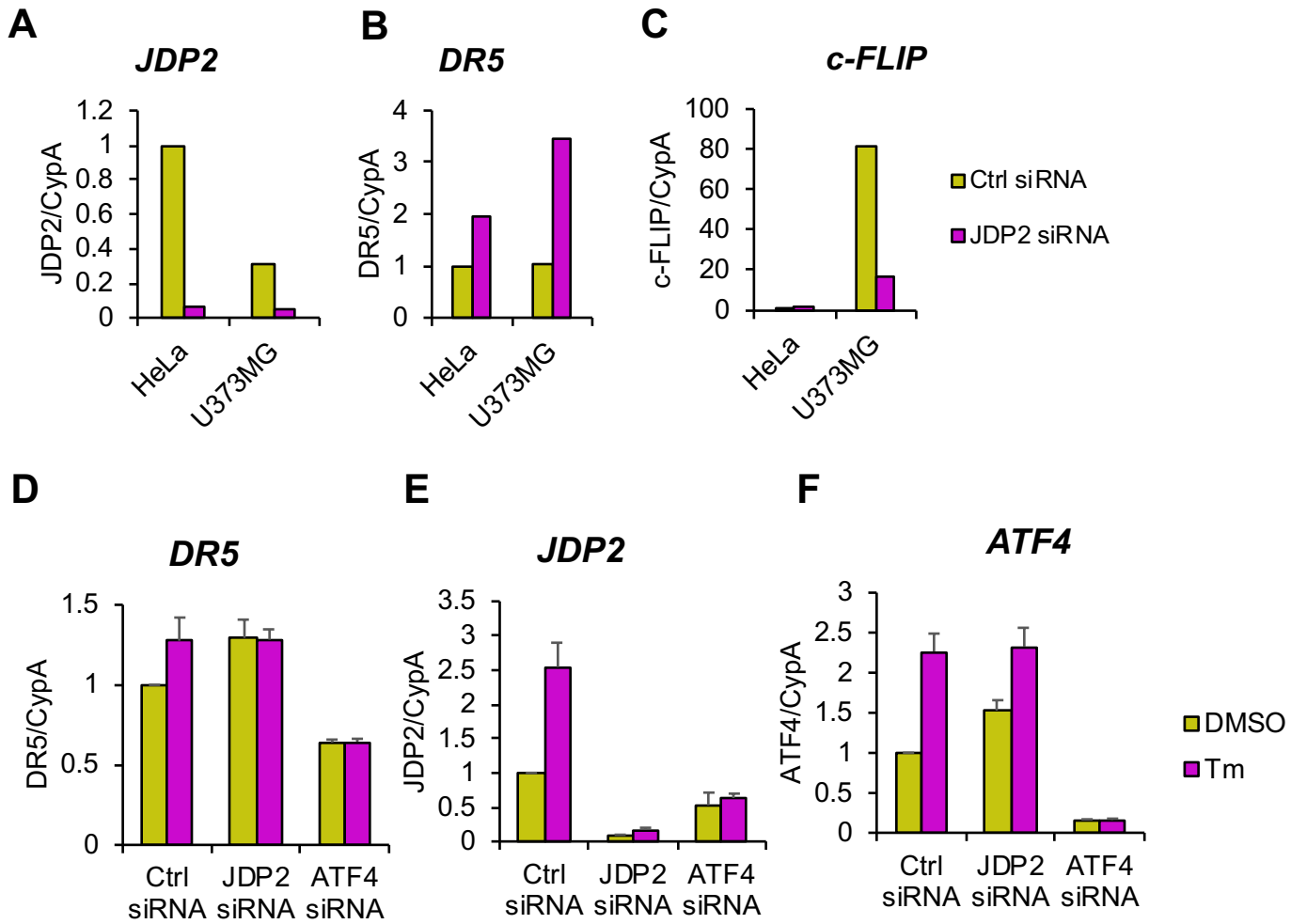

Supplement: Supplementary file 1 — Fig. S1. Comparison of gene expression in either control or JDP2 knockdown HeLa and U373MG cells. Gene expression for JDP2 (A), DR5 (B) and c‐FLIP (C) was determined by RT‐qPCR and normalized with cyclophilin A expression. (D to F) HeLa cells were transfected with either control or JDP2 or ATF4 siRNA. After 24 h incubation, the media was replaced and treated with DMSO or 1 μg/mL tunicamycin for 6 h. RT‐qPCR was performed to evaluate DR5 (D), JDP2 (E) and ATF4 (F) expression. The values are presented as mean ± SE of three independent experiments (n = 3). [file FEB4-10-2771-s001.pdf]

**A**

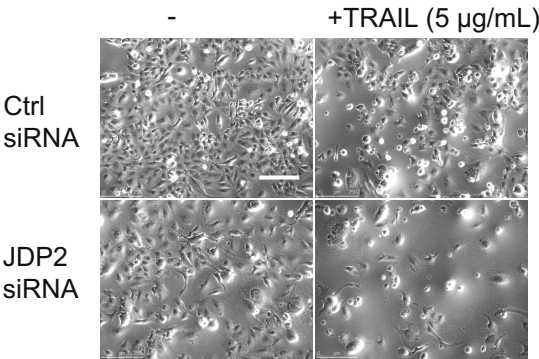

**B**

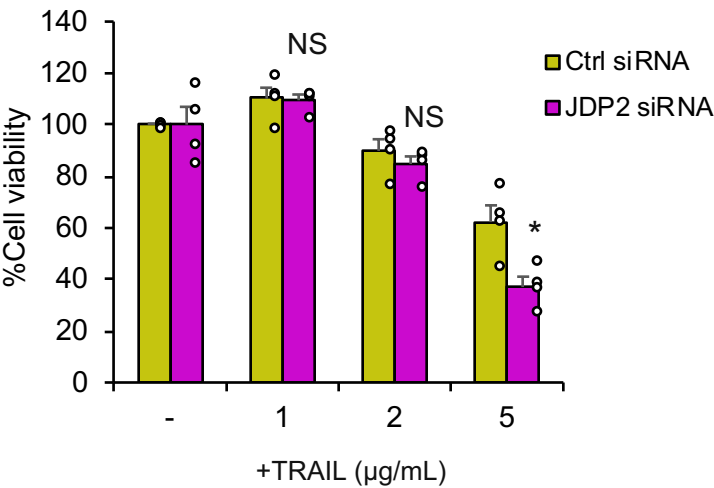

Supplement: Supplementary file 2 — Fig. S2. TRAIL sensitivity of T98G human glioma cells. T98G cells were transfected with either control or JDP2 siRNA by using Lipofectamine RNAiMAX. After 24 h transfection, the media was replaced with fresh media and incubated for 48 h in the absence or presence of TRAIL as indicated in figure. (A) Representative photo images of control or JDP2 knockdown cells treated with 5 μg/mL recombinant TRAIL for 48 h. Scale bar indicates 250 μm. (B) Cell viability was determined by CCK‐8 kit. The values are presented as mean ± SE of three independent experiments (n = 3) (‐TRAIL = 100%). The asterisks indicate a significant decrease compared with control (*, p < 0.05; NS, not significant) by Student’s t‐test. [file FEB4-10-2771-s002.pdf]
